# Detecting parent of origin and dominant QTL in a two-generation commercial poultry pedigree using variance component methodology

**DOI:** 10.1186/1297-9686-41-6

**Published:** 2009-01-05

**Authors:** Suzanne J Rowe, Ricardo Pong-Wong, Christopher S Haley, Sara A Knott, Dirk-Jan De Koning

**Affiliations:** 1 Roslin Institute and R(D)SVS, University of Edinburgh, Midlothian, EH25 9PS, UK; 2 Institute of Evolutionary Biology, University of Edinburgh, Kings Buildings, Edinburgh, EH9 3JT, UK; 3 Medical Research Council, Human Genetics Unit, Western General Hospital, Crewe Road, Edinburgh EH4 2XU, UK

## Abstract

**Introduction:**

Variance component QTL methodology was used to analyse three candidate regions on chicken chromosomes 1, 4 and 5 for dominant and parent-of-origin QTL effects. Data were available for bodyweight and conformation score measured at 40 days from a two-generation commercial broiler dam line. One hundred dams were nested in 46 sires with phenotypes and genotypes on 2708 offspring. Linear models were constructed to simultaneously estimate fixed, polygenic and QTL effects. Different genetic models were compared using likelihood ratio test statistics derived from the comparison of full with reduced or null models. Empirical thresholds were derived by permutation analysis.

**Results:**

Dominant QTL were found for bodyweight on chicken chromosome 4 and for bodyweight and conformation score on chicken chromosome 5. Suggestive evidence for a maternally expressed QTL for bodyweight and conformation score was found on chromosome 1 in a region corresponding to orthologous imprinted regions in the human and mouse.

**Conclusion:**

Initial results suggest that variance component analysis can be applied within commercial populations for the direct detection of segregating dominant and parent of origin effects.

## Introduction

Despite intense selection there is evidence to suggest that there is still much variation that might be exploited within commercial populations [1,2]. The effectiveness of selection procedures utilising genomic information can be increased by correctly identifying the mode of inheritance of desired variants. For example, Hayes and Miller [3] show that including dominance effects in mate selection can be a powerful tool for exploiting previously untapped genetic variation while Dekkers and Chakraborty [4] discuss maximization of crossbred performance by incorporating information from overdominant QTL.

Historically, much of the success in commercial poultry breeding and many other agricultural species has relied on utilizing heterosis and reciprocal effects [5-8], yet the underlying genetic architecture is still not clear. It appears that both maternal effects and dominant or over-dominant genes play a role [9]. Tuiskula-Haavisto and Vilkki [10] suggest that there is also recent evidence for the role of parentally imprinted mechanisms in poultry to explain the underlying mechanism for reciprocal effects.

Despite increasing evidence for parent of origin effects in crosses between divergent lines of poultry, imprinting in poultry remains a contentious issue.

Genomic imprinting affects many mammalian genes [11] and is brought about by epigenetic instructions or imprints that are laid down in the parental germ cells [12]. Imprinting is most prevalent in foetal development and until recently was considered best described by the parental conflict hypothesis [13]. In viviparous animals this occurs where the male exerts selection pressure for offspring to maximise use of maternal resources whereas the female limits this allocation of resources to preserve herself and future offspring. As there is no apparent parental conflict, the presence of imprinting was not thought to occur in oviparous species. Furthermore, IGF2 has been shown to be imprinted and expressed from paternal allele in man rabbit, mice, pig, and sheep [14,15], but not in the chicken [16]. There is, however, recent evidence for imprinted genes in birds and lower vertebrates and for shared orthologues with mammalian imprinted genes [17,18]. Different species may also have species specific imprinted genes [19]. Current theory suggests that the evolution of imprinted genes is a dynamic step-wise process with orthologues present on separate chromosomes before imprinting arose. These conserved orthologues were selected during vertebrate evolution becoming imprinted only as the need arose [18,20]. Lawton *et al*., [21] show that transcriptional silencing at imprinted loci has evolved along independent trajectories in mammals and marsupials. Imprinted genes are characteristically found in a clustered organization with 80% physically linked with other imprinted genes. These clusters are conserved in mammals, marsupials and flowering plants. [12]. Studies reporting QTL with parent of origin effects in chicken show a similar pattern tending to cluster on a few macrochromosomes with 78% of imprinted gene orthologues residing on chicken chromosomes 1, 3, and 5 [10,18].

Both dominant and imprinted QTL effects have been identified in poultry for economically important production and disease resistance traits. Ikeobi *et al*., [22] found that 1/3 of QTL found for fat related traits in a broiler-layer cross showed dominance effects; Yonash *et al*., [23] found both partial and overdominance QTL effects for resistance to Marek's disease, while Kerje *et al*., [24] and Tuiskula-Haavisto *et al*., [25] report dominant effects for egg production traits. Parent of origin effects in poultry are reviewed by Tuiskula-Haavisto *et al*., [10] and have been found for bodyweight, carcass and egg production traits [26-28].

All of these studies have involved crosses between lines or divergent populations, reviewed by Hocking [29] and Abasht *et al*.,[30]. Detection of QTL effects, however, within model organisms or experimental populations is costly and potentially of limited relevance to populations under selection. It is of much greater benefit to directly explore QTL segregating within commercial populations. A variance component or pedigree based approach can be applied to map QTL directly within the population under selection and by simple extension of genetic models can potentially also be used to dissect the mode of inheritance at the QTL. Here we use a variance component approach to look for dominant and imprinted QTL associated with bodyweight and conformation score measured at 40 days in a two-generation commercial broiler population.

## Methods

### Data

Phenotypes on conformation score and bodyweight, both measured at 40 days, were available for a commercial broiler dam line from Cobb Breeding Company Ltd. Conformation score is a subjective measure of fleshiness scored from 1–5 and was treated as normally distributed. A two-generation pedigree was available with a total of 2708 offspring with phenotypes and genotypes for markers in candidate QTL regions on chicken chromosomes 1, 4 and 5. Candidate regions were based on a previous three generation study of the Cobb population [1]. Forty-six sires were mated to 100 dams with an average of two dams per sire, 59 half sibs per sire and 27 full sibs per dam. The number of progeny per sires and dam ranged from 9 to 149 and 14 to 44, respectively. Birds were genotyped for markers spaced approximately every 16, 14 and 8 cM on chromosomes 1, 4, and 5, respectively. Markers were selected from the consensus linkage map [31]. Linkage maps were estimated using CriMap [32] and linkage groups corresponded to the consensus map at approximately 128–205 cM, 75 – 182 cM, and 57–104 cM for chicken chromosomes 1, 4 and 5 respectively. Marker distances and consensus map positions are given in additional file 1. Progeny were from two flocks across 17 hatch weeks. Fixed effects of sex, age of dam, and hatch within flock were fitted. Summary statistics and heritabilities can be found in Table 1. The correlation between the two traits was 0.34 (0.03). Further details can be found in Rowe *et al*. [33].

**Table 1:** Summary statistics and heritabilities for trait data

	Mean (min, max)	sd	h^2 ^(s.e.)	c^2 ^(s.e.)
Bodyweight (g)	2510 (820, 3560)	300.4	0.08 (0.06)	0.045 (0.03)
Conformation score	3.35 (1, 5)	0.83	0.08 (0.06)	0.03 (0.03)

h^2 ^polygenic heritability based on animal model, c^2 ^random common environmental or maternal effect

### Statistical genetic models for variance component analysis

Following a two-step approach similar to that described by George *et al*. [34], identical by descent (IBD) coefficients were estimated for all relationships in the pedigree to calculate the covariance matrices for the QTL effects, which were subsequently used in a linear mixed model.

### IBD Estimation

The **G, G**_**M**_, **G**_**P **_and **D **are the appropriate relationship matrices used to model the additive, maternal, paternal and dominant QTL effects at each position tested. They are conditional on flanking marker information and therefore unique for each position evaluated for a QTL. Here the matrices were calculated every 5 cM.

It can be shown that these relationship matrices are easily estimated from the gametic IBD matrix, a 2n × 2n matrix containing the probability of identity of descent between any of the two gametes of an individual with the gametes of the remaining individuals in the pedigree [25]. Where *P*_1 _is the paternally derived allele at a locus and *P*_2 _is the maternally derived allele elements of the gametic IBD matrix for individuals i and j at a single locus are $G_{ij}=\left[\begin{array}{cc} P_{11} & P_{12}\\ P_{21} & P_{22} \end{array}\right]$. From this the additive covariance between i and j is *r*_*ij *_= 1/2(*P*_11 _+ *P*_12 _+ *P*_21 _+ *P*_22_) and the covariance due to dominance i.e. the inheritance of two alleles identical by descent is *u*_*ij *_= *P*_11_*P*_22 _+ *P*_12_*P*_21 _[25]. The probability of individuals i and j sharing paternal or maternal QTL alleles IBD is simply *P*_11 _or *P*_22 _respectively.

In contrast to George *et al*. [34] who used a Monte-Carlo method, the gametic IBD matrix was estimated with the recursive method of Pong-Wong *et al*., [35] (software available on request from the author), which uses the closest fully informative or phase-known flanking marker to estimate the IBD at the putative QTL. Variance components for each model were estimated using REML [36] implemented in the ASReml package [37].

The statistical models used were:

(1) **y **= **Xβ **+ **Zu **+ **Wc **+ **e **(null or polygenic)

(2) **y **= **Xβ **+ **Zu **+ **Wc **+ **Za + e **(additive QTL)

(3) **y **= **Xβ **+ **Zu **+ **Wc **+ **Za **+ **Zd **+**e **(additive QTL + dominant QTL)

(4) **y **= **Xβ **+ **Zu **+ **Wc **+ **Z_m_m **+ **Z**_**p**_**p **+**e **(maternal QTL + paternal QTL)

(5) **y **= **Xβ **+ **Zu **+ **Wc **+ **Z_p_p **+**e **(paternal QTL)

(6) **y **= **Xβ **+ **Zu **+ **Wc **+ **Z_m_m **+**e **(maternal QTL)

where **y **is a vector of phenotypic observations, **β **is a vector of fixed effects, **u, a, d, m**, **p**, **c **and **e **are vectors of random additive polygenic effects, additive and dominance QTL effects, maternal and paternal QTL effects, maternal effects and residuals, respectively. **X, Z, W**, **Z**_**m**_, and **Z**_**p **_are incidence matrices relating to fixed and random genetic, direct maternal, maternally expressed, and paternally expressed QTL effects, respectively.

Variances for polygenic and QTL effects are distributed as follows: var(**u**) =**A**σ^2^_a_, Var(**a**) = **G**σ^2^_q_, Var(**d**) = **D**σ^2^_d_, Var(**m**) = **G**_**M **_σ^2^_m_, Var(**p**) = **G**_**P **_σ^2^_p_, var(**e**) = **I**σ^2^_e_. For the non-genetic maternal effect Var(**c**) = **I**σ^2^_c_. **A **is the standard additive relationship matrix based on pedigree data only and the relationship matrices **G, G**_**M**_, **G**_**P **_and **D **for a given QTL position are calculated from the gametic **IBD **matrix as outlined by Liu *et al*.,[38].

### Test statistic

A test statistic for a given location was obtained by comparing the likelihood of the full versus the reduced model. Twice the difference between the log likelihood of the full versus the reduced model was used as a log likelihood ratio test (LRT). Permutation was used to set significant thresholds. For linkage group-wise test statistics, genotypes were permuted within dam families to remove associations with IBD status and phenotype. Because permutation was done within dam families, i.e. sibs swap genotypes but retain phenotypes, the **A **matrix and therefore the estimated polygenic variance remained the same. After each permutation, analyses for all models were repeated for every test position along the chromosome and the highest test statistic was recorded. After 1000 permutations the test statistics were ranked and the 95th percentile used for a linkage group-wise 5% type 1 error rate. Separate permutation analyses were carried out for each trait. Permutation analysis for all three chromosomes was similar so thresholds were set using the results from chromosome 4 as this is the linkage group with the most tests. In each case the highest test statistic for each model was recorded regardless of position. Empirical thresholds for each test are given in Table 2. For plotting purposes test statistics for each position were converted to rank by comparison to the results of the permutation analysis, and the rank subsequently divided by 10. For example, a test statistic corresponding to the 950^th ^ranked value from the permutation analysis was plotted with a value of 95, corresponding to a 5% type 1 error.

**Table 2:** Tests for QTL effects and corresponding empirical thresholds for 5% type 1 error based on 1000 permutations

Test	QTL in Model		QTL effect tested for	Bodyweight	Conformation score
				
	alternative (H1)	null (H0)		*LRT (5%)	*LRT (5%)
*Add*	*add *(2)	null (1)	additive	5.74	4.53
*Addom*	*add + dom *(3)	null (1)	additive + dominant	6.98	5.84
*pat + mat*	*pat + mat *(4)	null (1)	paternal + maternal	3.05	2.94
*Pat*	*pat *(5)	null (1)	paternal	7.16	6.6
*Mat*	*mat *(6)	null (1)	maternal	5.38	4.54
*Dom*	*add + dom *(3)	*add *(2)	dominant	4.80	5.12
*Imp*	*pat + mat *(4)	*add *(2)	parent of origin	3.18	3.43
**patvfull	*pat + mat *(4)	*pat *(5)	maternally expressed	4.14	4.32
**matvfull	*pat + mat *(4)	*mat *(6)	paternally expressed	4.5	3.58

* LRT is the chromosome-wise empirical threshold for 5% type 1 error rate for test statistic (twice the difference between log for the alternative and null models), estimated by 1000 iterations.

** For example, if the test of *patvfull *is significant the model incorporating paternal and maternal QTL is explaining more variation than the paternal QTL indicating some level of maternal expression. If there is no significant difference between the *pat + mat *model and *mat *model the maternal QTL is explaining all the variation.

#### Detection of dominant QTL effects

To detect dominant QTL effects, three tests were carried out:

(i) *add*, comparing the additive QTL model (2) versus the null model (1) to test significance of the QTL variance component under a purely additive model;

(ii) *addom*, comparing the additive QTL + dominance QTL model (3) versus the null model (1) to test significance of QTL variance components under a model including additive and dominance effects;

(iii) *dom*, comparing the additive QTL + dominance QTL model (3) vs. the additive QTL model (2) to test the significance of the dominance variance component.

Tests (i) and (ii) are used in the initial search for the QTL whereas test (iii) is applied subsequently to test specifically for the dominance component. The *dom *test was applied at all positions regardless of significance of other tests.

#### Parent of origin effects

Initially QTL can be searched for using additive (*add*), *pat + mat *or single parental models (*mat *or *pat*). To test for imprinting four tests were carried out at each position:

(i) *pat + mat*, comparing the paternal QTL + maternal QTL model (4) vs. the null model (1) to test the significance of an additive QTL whilst allowing the maternal and paternal components to vary;

(ii) *imp*, comparing the *pat + mat *model (4) vs. the *add *model (2) to test whether the additive effect was better explained by allowing different parental contributions;

(iii) *patvfull*, comparing the paternal QTL model *pat *(5) vs. the *pat + mat *model to test for contribution of a paternally inherited QTL to the QTL variance;

(iv) *matvfull*, comparing the paternal QTL model *mat *(6) vs. the *pat + mat *model to test for contribution of a maternally inherited QTL to the QTL variance.

Again, all tests were carried out at all positions regardless of significance of other tests. Following Hanson *et al*., [39] under an additive model both parents contribute equally whereas for an imprinted QTL only one parent is expected to show expression. For example, for a maternally expressed QTL the expectation is that the *patvfull *test is significant and the *matvfull *test is not significant. For non-imprinted QTL the expectation is that both tests are significant because there is expression from both parents.

### Maternal effect

Common environment effects are often, at least partially, confounded with dominance and imprinting as shown by Rowe *et al*., [40] thus common family environment or 'dam' effects were included in all models.

## Results

Table 1 gives heritabilities for the two traits. These are low, probably due to selection of the parents [41]. Because heritability estimates are based on the contrast of between and within family variance and QTL variance is based mainly on within family variance low trait heritability is not expected to affect QTL detection.

### Additive and dominant QTL effects

**Figure 1:**
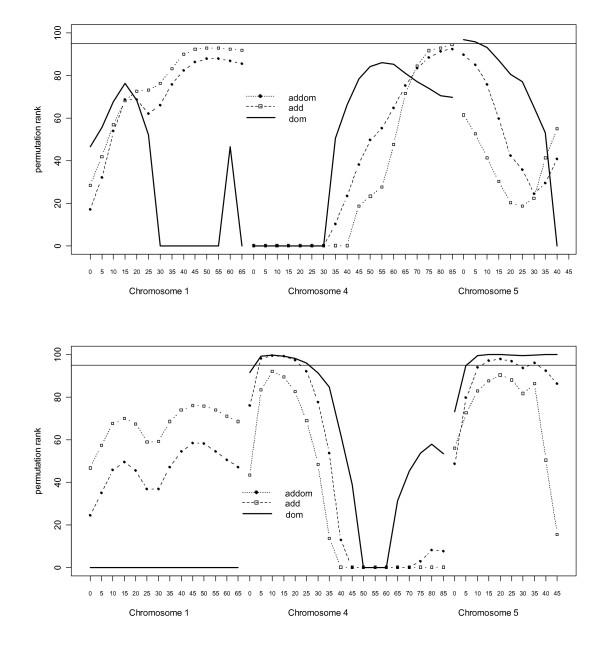
**Interval mapping of additive and dominant QTL effects on chicken chromosomes 1, 4 and 5 for weight (top) and conformation-score (bottom)**. The Y-axis shows the scaled rank of the test statistic obtained when compared to 1000 permutations of genotype within dam for 18 positions on chromosome 4 for weight and conformation-score. Test *add *is rank of test statistic obtained for model testing for additive QTL, *addom *is test statistic obtained from testing for both additive and dominant QTL effects and *dom *is test between two models for dominance only. Dam effect was fitted. Solid line at top is 5% empirical linkage group-wise significance

Figure 1 shows QTL effects under additive and dominant QTL models for bodyweight and conformation score. There were chromosome-wide significant dominant QTL effects for conformation score on chromosomes 4 and 5. These effects were considerable, explaining 6.2 and 4.5% of the phenotypic variance, respectively. Table 3 shows that the dominant QTL explains all of the QTL variance (i.e. the estimated additive effect of the QTL is zero when a model with both an additive and dominant QTL effect is fitted).

**Table 3:** Highest test statistics and proportion of phenotypic variance explained at most likely QTL position when fitting additive QTL and dominance QTL effects for 40-day bodyweight and conformation score on chicken chromosomes 1, 4 and 5

Chr	pos	Model fitting additive QTL					Model fitting additive and dominant QTL						
		^††^LRT	^†^variance component				^††^LRT		^†^variance component				
		*add*	Va	Vp	Vc	res	*addom*	*dom*	Va	Vp	Vc	Vd	res
Bodyweight													
1	55	5.0	0.07	0.09	0.02	0.89	5	0	0.07	0.01	0.02	0.00	0.89
4	85	5.0	0.04	0.04	0.03	0.89	5.7	0.6	0.03	0.05	0.02	0.02	0.88
5	5	1.4	0.02	0.06	0.02	0.89	5.3	3.9*	0.00	0.08	0.01	0.05	0.86
Conformation score													
1	50	2.3	0.04	0.04	0.04	0.89	2.3	0	0.04	0.04	0.04	0.00	0.88
4	15	4.1	0.04	0.04	0.05	0.87	10.4*	6.3*	0.00	0.06	0.03	0.06	0.84
5	25	3.8	0.03	0.04	0.04	0.87	7.9*	8.1*	0.00	0.07	0.04	0.04	0.85

^†^Proportion of phenotypic variance explained at highest test statistic (LRT) Vp: polygenic variance, Va: additive QTL variance, Vc: maternal (dam) variance, Vd: dominant QTL variance, res: residual variance

^††^LRT is test statistic obtained from best position (pos), *add *is additive QTL versus null model, *addom *is additive and dominant QTL versus null model, *dom *is additive and dominant QTL versus additive QTL model * 5% linkage group-wise significance calculated from 1000 permutations of within dam genotype for 18 positions on chromosome 4 for weight and conformation-score

### Parent-of-origin QTL effects

**Figure 2:**
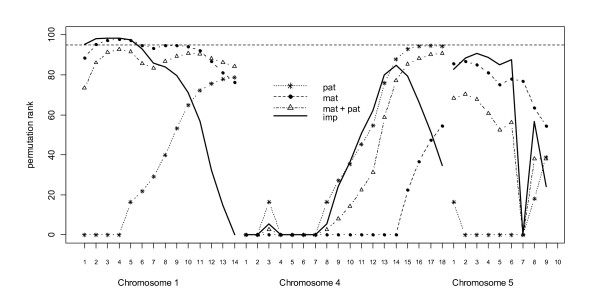
**Interval mapping of parent of origin QTL effects for body-weight on chicken chromosomes 1, 4 and 5**. The Y-axis shows the scaled rank of the test statistic obtained when compared to 1000 permutations of genotype within dam for 18 positions on chromosome 4 for conformation score. *Mat *and *pat *are testing for maternally or paternally expressed QTL respectively. *Mat *+ *pat *is fitting both maternal and paternal expression and *imp *is testing difference between *add *model versus *mat + pat *model. Dashed line at top is 5% empirical linkage group-wise significance

Figure 2 shows rank of test statistics when compared to permutation analysis for bodyweight on chromosomes 1, 4 and 5. Figure 1 shows that there was not significant evidence for a purely additive QTL at the beginning of the chromosome 1. Figure 2, however, shows that the *pat + mat *model is significantly better than the *add *model and there is evidence for a maternally expressed QTL on chromosome 1. Table 4 also shows that the *patvfull *test is significant whereas the *matvfull *test is not indicating maternal expression. Furthermore, all of the QTL variance is explained by the maternal QTL (Table 5).

**Table 4:** Test statistics for all models at highest test statistic for separate parental QTL contributions

Chr	Pos (cM)	Model/Test^†^								
		*add*	*addom*	*pat+mat*	*pat*	*mat*	*Imp*	*patvfull*	*matvfull*	*dom*
Bodyweight										
1	10	1.7	2.6	6.3	0.0	6.3**	4.6*	6.3*	0.0	0.8
4	85	5.0	5.7	5.6	5.3	0.6	0.6	0.3	5.0*	0.6
5	5	1.0	4.4	3.2	0.0	3.2	2.2	3.2	0.0	3.4*
Conformation score										
1	10	1.8	1.8	5.4	0.0	5.4*	3.6*	5.4*	0.0	0.0
1	65	1.9	1.9	4.0	4.0	0.0	2.1	0.0	4.0*	0.0
4	10	4.1	10.4*	4.4	2.1	2.6	0.2	2.3	1.8	6.3*
4	85	0.1	0.5	2.6	0.0	2.6	2.5	2.6	0.0	0.4
5	30	2.8	5.7	5.5	0.1	5.4*	2.7	5.5*	0.2	5.7*

* and ** indicate 5 and 2.5% chromosome wise significance under permutation analysis

† separate parental contributions modelled by comparing a *pat + mat *model fitting separate maternal and paternal QTL effects versus no QTL (null model), add is additive QTL versus null model, addom is additive and dominant QTL versus null model, *dom *is additive and dominant QTL versus additive QTL model, *mat *and *pat *are maternal and paternal QTL models versus null respectively, imp test is *pat + mat *model versus *add *model.

**Table 5:** Proportion of phenotypic variance explained by polygenic, dam, paternal QTL and maternal QTL effects fitted in a *pat+mat *model at the position of the highest test statistic for *pat+mat *model versus no QTL

Chr	Position (cM)	Variance component			
		polygenic	dam	pat QTL	mat QTL
Bodyweight					
1	10	0.09	0.00	0.00	0.06*
4	85	0.03	0.04	0.03	0.01
5	5	0.09	0.01	0.00	0.04
Conformation score					
1	10	0.08	0.03	0.00	0.05
1	65	0.05	0.06	0.02	0.00
4	10	0.05	0.05	0.02	0.03
4	85	0.08	0.04	0.00	0.03
5	30	0.08	0.04	0.00	0.04

The table shows the proportion of phenotypic variance explained by variance components. In the null model with no QTL, fitted polygenic heritability is 0.08, and dam component (Vc) estimated at 0.05 for conformation score and 0.03 for bodyweight.

**Figure 3:**
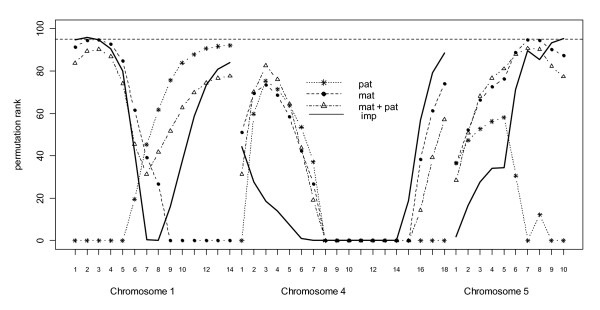
**Interval mapping of parent of origin QTL effects for conformation-score on chicken chromosomes 1, 4 and 5**. The Y-axis shows the scaled rank of the test statistic obtained when compared to 1000 permutations of genotype within dam for 18 positions on chromosome 4 for conformation score. *Mat *and *pat *are testing for maternally or paternally expressed QTL respectively. *Mat *+ *pat *is fitting both maternal and paternal expression and imp is testing difference between *add *model versus *mat + pat *model. Dashed line at top is 5% empirical linkage group-wise significance

Figure 3 shows test statistics for conformation score on chromosomes 1, 4 and 5. For chromosomes 1 and 5 there is some evidence for a maternally expressed QTL affecting conformation score although the *imp *test is only significant for chromosome 1. Chromosome 4 has two linkage peaks, however neither reaches significance.

## Discussion

Dominant and parentally expressed QTL effects were distinct from one another and do not appear to be confounded therefore they are discussed separately.

### Chromosome 1

There is suggestive evidence for a maternally expressed QTL on chromosome 1 for both weight and conformation score associated with marker interval ADL0307-LEI0068, a region orthologous with imprinted regions in the mouse and human associated with Prader-Willi/Angelman syndrome [42]. This region of chromosome 1, corresponding to approximately 128 to 151 cM on the consensus map, is within a marker interval associated with many fat and carcass traits in chickens [22,24,30,43,44]. Furthermore, McElroy *et al*., [28] and Tuiskula-Haavisto *et al*., [26] both find maternally expressed QTL within the same marker bracket associated with egg production. Sharman *et al *[27] find imprinted effects for skeletal traits at 135 cM on chromosome 1. Tuiskula-Haavisto *et al*., [26] also find a paternally expressed QTL associated with age at first egg in the same marker interval as the putative paternally expressed effect seen here for conformation score.

When a common environment or dam effect was omitted from the model (results not shown) evidence, in particular, for the maternally expressed QTL on chromosome 1 increased. The *mat + pat *and *imp *tests also reached significance if a dam effect was not accounted for. This is possibly due to confounding of effects i.e. common environment can give spurious variance at the QTL and further highlights the importance of fitting maternal effects to avoid spurious detection of QTL. De Koning *et al *[1] found significant additive effects for bodyweight and conformation and a strong dam effect associated with this region using a three generation design from the same population. This could indicate that a strong component of the effect on chromosome 1 associated with bodyweight and conformation score comes from maternally influenced egg traits. Maternal influence on fresh egg weight and subsequent bodyweight, particularly early growth is well documented [45-47]. Kerje *et al *[24] report a strong correlation between egg weight and adult bodyweight (r = 0.62, 0.0001) and a QTL for growth at the beginning of chromosome 1 explaining half the phenotypic variation seen in egg weight.

### Chromosome 4

There appear to be two separate QTL segregating for bodyweight and conformation score on chromosome 4. For bodyweight there is an additive QTL in the region of ADL0266 - LEI0076 as found by Kerje *et al*., [24] and Jacobsson *et al*., [48]. There is greater evidence for this from the paternal analysis. Although the paternal QTL appears to explain most of the additive variance there is insufficient evidence for imprinting i.e. the test of the *pat + mat *model versus an additive model is not significant. For conformation, a dominant and potentially over-dominant QTL explaining all of the QTL variance maps to around 80–118 cM on the consensus map. Yonash *et al*., [23] find partial and overdominance for QTL affecting resistance to Marek's disease in this marker bracket. Although Ikeobi *et al *[44] find many dominant effects for carcass trait QTL, they find the QTL on chromosome 4 tends to behave additively as a single locus affecting many traits. Sharman *et al *[27] report QTL for many traits associated with skeletal traits on chromosome 4 including a dominant QTL associated with tibial marrow diameter at ADL0266-ROS0024.

### Chromosome 5

On chromosome 5 there appear to be dominant effects for bodyweight and conformation traits. Although the test for dominance (*dom*) is significant for bodyweight the actual QTL does not reach linkage group-wise significance. Ikeobi *et al*., [44] also found modest dominance effects for growth traits in this region. For conformation score, there is evidence for most of chromosome 5 for a significant dominant QTL and maternal expression at the end of the linkage group. Abasht *et al*., [49] also find a maternal sex interaction with fat traits in this marker bracket. Chromosome 5 has been associated with many paternally expressed traits [27,28] and although the linkage group does not span the region, the first marker interval is close to a conserved gene cluster of twelve imprinted gene orthologues shown to replicate asynchronously. Despite this, here we see no evidence for paternal imprinting on chromosome 5. Ikeobi *et al*., find many QTL for traits associated with weight and carcass composition in this region although little dominance and no imprinting.

### General discussion

Given that we are only using a two-generation pedigree we have insufficient evidence to confirm that these are truly imprinted effects, only that statistically there is evidence for uniparental expression. Heuven *et al*., [50] show that spurious imprinted effects can be detected due to differences between the number of QTL alleles or haplotypes segregating in sires and dams. This can occur for a number of reasons, for example; too few sires or dams included in the analysis, different genetic backgrounds leading to different QTL allele frequencies in sires and dams and or differing amounts of LD generated between QTL alleles and markers.

To ensure information on a putative QTL is available from both parents there is a requirement for enough sires and dams to ensure segregation together with enough offspring to detect QTL. Furthermore the QTL allele frequency should be roughly equal in sires and dams. Here, these requirements are satisfied by using a large number of sires and dams. Furthermore, because the analysis took place within a broiler dam line, i.e. sires and dams have the same genetic background, neither differing allele frequencies due to parental origins or sampling issues are likely causes of spurious imprinting. Marker allele frequencies are not significantly different between sires and dams and an average of 24 sires and 25 dams are informative at any given marker (results not shown).

It is possible that differences in LD between the marker and QTL alleles might occur if parents originate from different populations, however again this is not the case here and furthermore, marker spacing makes it unlikely that strong LD in one sex could have caused differences in variation as it has been shown that linkage disequilibrium in commercial poultry populations rarely exceeds 1 or 2 cM [51]. Using simulation, Tuiskula-Haavisto *et al*., [26] also concluded that segregation differences are an unlikely source of spurious parent-of-origin effects.

A further source of error might be spurious detection of maternally expressed QTL due to common maternal environment, here common environment is fitted within the linear model. Finally, it is feasible that there are many QTL causing a complex inheritance pattern although again due to sires and dams coming from the same lines it is unlikely that different QTL would be segregating. It would be difficult to test this using the current structure due to the complexity of the analysis as it is unlikely that the extra number of variance components added could be successfully estimated. It is also unlikely that enough information could be derived from the marker spacing to estimate multiple QTL within discrete confidence intervals.

Further evidence for the results found here is that imprinted effects on chromosome 1 were found in regions previously identified as parentally expressed in poultry and orthologous with genome-imprinted regions in humans and mice.

### Testing strategy

Testing many models at each position raises its own multiple testing issues, one strategy might be to only carry out subsequent testing after identifying a significant additive QTL. This, however, can lead to QTL being missed due to the use of an inappropriate model. When testing for dominance the dominant QTL on chromosome 4 would not have been detected under an additive model.

Similarly for parentally expressed QTL, it follows that the contrast may not be greatest at the highest test statistic for the *pat + mat *or *add *models but at the highest test statistic for the individual parental QTL i.e. *mat *or *pat*. For example, on chromosome 5 the greatest evidence for a maternal QTL and for the *imp *test is not at the same position as the highest test statistic for a search under the *pat + mat *model versus null. On chromosome 1, there is a maternal QTL and the *imp *test is significant, however the *pat + mat *model is not. The *pat + mat *model versus null is perhaps diluted by the non expression from the imprinted parent as it is explaining the same amount of variation with an extra degree of freedom. Here we also find that a bodyweight QTL on chromosome 4 could be declared as paternally expressed based upon separate parental QTL models but there is insufficient evidence when comparing a Mendelian versus a *pat + mat *or imprinted model. It is difficult to know whether this is due to information source, or perhaps too stringent a threshold on the *imp *test or too lenient on the *pat *test.

## Conclusion

A large dominant and potentially over-dominant QTL for conformation score is segregating on chicken chromosome 4. This QTL is also detected under an additive model. However, the additive variance becomes zero in a model that also fits a dominance component. There is also evidence for dominant QTL affecting bodyweight and conformation on chromosome 5. There is suggestive evidence for a paternally imprinted or maternally expressed QTL affecting bodyweight and conformation score on chromosome 1 in a region orthologous with human and mouse imprinted regions and close to previously reported imprinted QTL affecting bodyweight and maternal traits in poultry. Initial results suggest that variance component analysis can be applied within commercial populations for the direct detection of segregating dominant and parent of origin effects.

## Competing interests

The authors declare that they have no competing interests.

## Authors' contributions

SJR carried out all of the data analysis, wrote and prepared the manuscript for submission, RP-W wrote the rtools software and aided with data analysis, DJK led the project, wrote the FORTRAN program incorporating the software and evaluated initial rounds of the manuscript. SJR, DJK, RP-W, CSH and SAK were involved in experiment design, critical evaluation and final manuscript revision. All authors read and approved the final manuscript.

## Supplementary Material

Additional file 1 **Appendix 1. Marker distances and consensus map positions.** Click here for file
