# Supplementary material for: Detecting parent of origin and dominant QTL in a two-generation commercial poultry pedigree using variance component methodology
Source: Genet Sel Evol. 2009 Jan 5;41(1):6. doi: 10.1186/1297-9686-41-6 (PMC2637028; doi:10.1186/1297-9686-41-6)
Supplement: Additional file 1 — Appendix 1. Marker distances and consensus map positions. [file 1297-9686-41-6-S1.doc]

|  |  | Size of interval (cM) | | | |  |
| --- | --- | --- | --- | --- | --- | --- |
| Marker Interval | Chr | Female | Male | Sex averaged | Consensus | Position consensus (cM) |
| *ADL0307-LEI0068* | 1 | 19 | 15.3 | 17.1 | 23 | 128-151 |
| *LEI0068-MCW0297* | 1 | 2.6 | 7.7 | 10.1 | 11 | 151-162 |
| *MCW0297-MCW0112* | 1 | 35.8 | 38.6 | 37.4 | 42 | 162-205 |
| Total linkage group | 1 | 57.4 | 61.6 | 64.6 | 76 |  |
| *ADL0241-ROS0015* | 4 | 6 | 8.9 | 7.6 | ? | ? |
| *ROS0015-ADL0194* | 4 | 24.3 | 27.2 | 25.5 | 38 | 80-118 |
| *ADL0194-MCW0085* | 4 | 9.1 | 7.6 | 8.2 | 2 | 118-120 |
| *MCW0085-ADL0266* | 4 | 14.3 | 10.7 | 12.5 | 17 | 120-137 |
| *ADL0266-LEI0076* | 4 | 31.3 | 32.5 | 31.9 | 45 | 137-182 |
| Total linkage group | 4 | 85 | 86.9 | 85.7 | 102 |  |
| *MCW0090-ROS0013* | 5 | 23.6 | 19 | 21.4 | 21 | 57-78 |
| *ROS0013-ADL0292* | 5 | 9.2 | 5.5 | 7.4 | 5 | 78-83 |
| *ADL0292-ROS0084* | 5 | 8 | 5.8 | 6.8 |  | ? |
| *ROS0084-ADL0023* | 5 | 3.3 | 9.1 | 6.5 |  | ? (77-104) |
| Total linkage group | 5 | 44.2 | 39.4 | 42.1 |  |  |
